# Partial amelioration of a chronic cigarette-smoke-induced phenotype in mice by switching to electronic cigarettes

**DOI:** 10.1007/s00204-025-04055-7

**Published:** 2025-04-18

**Authors:** Alexander N. Larcombe, Emily K. Chivers, Katherine R. Landwehr, Luke J. Berry, Emma de Jong, Rachel R. Huxley, Arthur Musk, Peter J. Franklin, Benjamin J. Mullins

**Affiliations:** 1 Respiratory Environmental Health, Wal-yan Respiratory Research Centre, The Kids Research Institute Australia, 15 Hospital Avenue, Nedlands, Perth, WA 6009 Australia; 2 Occupation, Environment and Safety, School of Population Health, Curtin University, Perth, WA Australia; 3 School of Human Sciences, The University of Western Australia, Perth, WA Australia; 4 Centre for Health Research, The Kids Research Institute Australia, The University of Western Australia, Perth, WA Australia; 5 The George Institute for Global Health, University of New South Wales, Sydney, NSW Australia; 6 Faculty of Health, Deakin University, Geelong, VIC Australia; 7 School of Population and Global Health, University of Western Australia, Perth, WA Australia

**Keywords:** E-cigarette, Cigarette smoke, Mouse, Lung health

## Abstract

Electronic cigarettes (“e-cigarettes”) are often marketed as smoking cessation tools and are used by smokers to reduce/quit cigarette smoking. The objective of this study was to assess the health effects of switching to e-cigarettes after long-term smoking in a mouse model and compare these effects with continued smoking, or quitting entirely. Adult BALB/c mice were whole-body exposed to mainstream cigarette smoke (2 h/day, 5 days/week) for 12 weeks prior to switching to flavoured e-cigarette aerosol (50:50 propylene glycol and glycerine) containing 18 mg/mL nicotine (2 h/day and 5 days/week), continuing cigarette smoking (2 h/day and 5 days/week), or quitting entirely for an additional 2 weeks. We then assessed a range of respiratory health outcomes including lung function and structure, pulmonary inflammation and changes in gene expression in the lung. Switching to e-cigarettes led to improvements in some aspects of respiratory health in mice compared with continued smoking, such as reduced neutrophilic inflammation in the lung. However, total cellular lung inflammation was still elevated and lung function was still impaired, in terms of airway responsiveness to methacholine, for e-cigarette use compared with quitting. Larger effects were typically seen in female mice compared to male. This study shows that switching to e-cigarettes after long-term cigarette smoking leads to improvements in some aspects of respiratory health, such as neutrophilic inflammation and the volume dependence of lung function compared with continued smoking. However, switching to e-cigarettes was not as effective as quitting smoking entirely.

**Supplementary Information:**

The online version contains supplementary material available at 10.1007/s00204-025-04055-7.

## Introduction

Tobacco smoking continues to be one of the world’s largest public health threats, accounting for approximately 8 million preventable deaths per year (WHO 2023b). It is a major cause of chronic-obstructive pulmonary disease (COPD) which accounts for almost half of these deaths (WHO 2023a), plus a range of other serious ailments. This global issue has motivated quit-smoking campaigns and the promotion of less harmful options for nicotine delivery such as nicotine replacement therapy (NRT) (McNeill et al. 2021). Accordingly, electronic cigarettes (e-cigarettes) have attracted considerable attention, and debate, around their efficacy as a potential tobacco harm reduction option (Bhatnagar et al. 2019; Britton et al. 2020; Bush et al. 2020; Giovacchini et al. 2022; Hartmann-Boyce et al. 2022). Unlike tobacco cigarettes, e-cigarettes heat and aerosolise a liquid (“e-liquid”) primarily consisting of propylene glycol, glycerine, nicotine, and flavourings (Klager et al. 2017; Larcombe et al. 2017). The aerosol produced is inhaled by the user. The lack of combustion means that e-cigarettes are often touted as a less harmful alternative to tobacco smoking (Bhatt et al. 2020; Löhler and Wollenberg 2019), although they are clearly not harm free (Banks et al. 2023; Bolt 2024; Bozier et al. 2020).

Despite limited evidence of their efficacy in helping people quit smoking, their use in this capacity is increasing (McNeill et al. 2021). What remains largely unknown is whether exclusively switching to using e-cigarettes after long-term cigarette smoking provides substantial health improvements over continued smoking and/or over “quitting” entirely. Unfortunately, most key studies in this space have been conducted by research groups with clear conflicts of interest (Kumar et al. 2021; Phillips et al. 2015; Polosa et al. 2016b, 2018). Unsurprisingly, such studies find that partial or complete switching to e-cigarettes in either COPD patients (Polosa et al. 2016b, 2018) or in pre-clinical models (Kumar et al. 2021; Phillips et al. 2015) results in substantial improvements in a range of health outcomes. These include reductions in COPD exacerbations, improved ability to perform physical activities (such as the 6-min walk test) and improved COPD Assessment Test (CAT) scores in COPD patients (Polosa et al. 2016a, 2018) in addition to reversal of inflammatory responses, and less dysregulation of the lung and nasal tissue transcriptome in pre-clinical models (Kumar et al. 2021; Phillips et al. 2015). Conversely, a number of recent studies have shown that e-cigarette aerosols elicit similar levels of toxicity, inflammation and lung damage as cigarette smoke when cells from COPD patients are exposed in vitro (Bozier et al. 2019; O’Farrell et al. 2021).

While it is evident that e-cigarette aerosols contain lower levels of many of the toxic components of cigarette smoke (Dusautoir et al. 2021), they also contain a range of chemicals not found in tobacco smoke. For many of these (which are primarily flavouring chemicals and excipient degradation products), the potential effects of heating and inhaling them remain largely unknown. Understanding these effects is crucial as certain e-cigarette flavouring chemicals (e.g. cinnamaldehyde) have known toxic effects (Clapp et al. 2017) and have thus been banned in some jurisdictions (TGA 2021). Combined with uncertainties raised by previous studies, this complexity means that a gap in our knowledge still exists in this space. Therefore, this study aimed to assess the measurable health effects of switching to e-cigarette use, compared with continued or quitting smoking, in a mouse model of long-term cigarette smoke exposure. We hypothesised that switching to e-cigarettes after long-term cigarette smoke exposure would have positive effects on respiratory health (compared with continued cigarette smoking), however these would not be as beneficial as quitting cigarettes entirely.

## Materials and methods

### Animals and exposure protocol

This project was approved by the Telethon Kids Institute Animal Ethics Committee (approval #323). All procedures were carried out in accordance with the Australian Code for the Care and Use of Animals for Scientific Purposes 8 th Edition (2013).

7-week-old male and female BALB/cARC mice (*n* = 12 per sex per treatment) were obtained from the Animal Resources Centre (Murdoch, WA, Australia) and housed under a 12-h light/dark cycle (light 0630-1830). Mice were housed in same-sex groups of four in individually ventilated cages (Sealsafe, Tecniplast, Buguggiate, Italy) on non-allergic, dust-free bedding. Mice had ad libitum access to an allergen-free diet (Specialty Feeds, Glen Forrest, WA, Australia) and water.

From 8 weeks of age (after a week of acclimation to the facility), the mice were exposed to tobacco cigarette smoke for two 1-h sessions per day (~ 9 am and ~ 3 pm), 5 days per week for 12 weeks to induce a chronic smoking/COPD phenotype (Larcombe et al. 2022). At the conclusion of the 12-week period, mice either continued tobacco smoke exposures (SS = smoke/smoke), switched to e-cigarette aerosol (SE = smoke/e-cigarette) or medical air (SA = smoke/air) exposures for a further 2 weeks, representing a smoker’s options to continue smoking, switch to using e-cigarettes, or quit nicotine/tobacco products all together respectively. Mice were weighed at least weekly during the exposure period.

All mice were whole-body exposed in separate compartments of a 27-L exposure chamber containing perforated dividers. The mice were placed in different positions in the chamber for each session to ensure consistent exposure between subjects. Separate chambers were used for air, smoke and e-cigarette exposures and these chambers were thoroughly cleaned between each exposure period. While whole-body exposure allows for oral uptake of excipient/nicotine through grooming, any off-target effects from this exposure route are likely to be negligible (Torrens et al. 2023) compared to the magnitude of the direct respiratory effects that are the focus of this study. Additionally, nose-only exposure to smoke has ethical implications and has been banned in some jurisdictions (NHMRC 2023).

### Tobacco smoke exposure: phenotype induction and “continue smoking” treatment

Tobacco smoke exposure was conducted as described previously (Larcombe et al. 2017). Briefly, a cigarette smoking machine (inExpose, SCIREQ^®^, Montreal, Canada) produced ISO standard 35 mL puffs of mainstream smoke once per minute. Six Winfield Red cigarettes (≤ 16 mg of tar, ≤ 1.2 mg of nicotine, Philip Morris, Melbourne, Australia) were used for each exposure session (total of 12 cigarettes per day). Throughout exposure, medical air flowed through the system at 2 L/min to evenly distribute the smoke and ensure oxygen levels remained adequate. All mice were subject to cigarette smoke as described above for a total of 12 weeks. At the conclusion of this period, one third of mice continued cigarette smoke exposure for an additional 2 weeks (SS treatment).

### Electronic cigarette aerosol exposure: “switch” treatment

After 12 weeks of tobacco smoke exposure, one-third of mice switched to being exposed to e-cigarette aerosol (SE treatment) for a further 2 weeks. An Innokin MVP4 e-cigarette with a SCION tank and 0.28 Ω coil (Innokin Technology Co. Ltd, Shenzhen, China) was used to generate 35 mL puffs of e-cigarette aerosol once per minute (total of 60 puffs per session and 120 puffs per day). The tank was filled with e-liquid at the beginning of each session and was topped up throughout the session as required to ensure the atomiser remained saturated. E-liquid was prepared in our laboratory using commercially available and laboratory grade ingredients. Commercial e-liquids (50:50 propylene glycol:glycerine; “berry” and “butterscotch tobacco” flavours) were combined and laboratory grade nicotine (Sigma Aldrich) was added to achieve a final concentration of 18 mg/mL nicotine. The excipient mix, nicotine level, and flavourings were chosen to represent common e-liquids used by adult e-cigarette users (Zare et al. 2018). Throughout exposure, medical air flowed through the system at 2 L/min to evenly distribute the aerosol and ensure oxygen levels remained adequate.

### Medical air exposure: “quit” treatment

After 12 weeks of tobacco smoke exposure, one-third of mice stopped tobacco smoke exposure and were exposed to medical air (SA treatment) for a further 2 weeks. Air was delivered at a rate of 2 L/min for two 1-h sessions per day, five days per week for 2 weeks.

### Lung function assessment

#### Animal preparation

24 h after the last exposure, all mice were prepared for lung function assessment as previously described (Larcombe et al. 2008). Briefly, the mice were anaesthetised with an intraperitoneal injection of 40 mg/mL ketamine and 2 mg/mL xylazine (0.01 mL/g body weight; Troy Laboratories, NSW, Australia). Once a surgical plane of anaesthesia was established, mice were tracheostomised and a polyethylene cannula (internal diameter = 0.086 cm, length = 1.0 cm) was inserted into the trachea and secured with suture.

#### Thoracic gas volume, lung mechanics and responsiveness to methacholine

Thoracic gas volume (TGV) and lung mechanics were assessed as previously described (Larcombe et al. 2011a, 2017). Briefly, the mice were ventilated at 400 breaths/min, with a tidal volume of 8 mL/kg and 2 cmH_2_O of positive-end expiratory pressure (PEEP) (Hugo Sachs Harvard Elektronik, March-Hugstetten, Germany). TGV was measured by electrically stimulating the intercostal muscles (six 3-ms, 20-V pulses) which induced inspiratory efforts against an occluded airway. Tracheal pressure and plethysmograph pressure were recorded and TGV calculated using Boyle’s Law (Jánosi et al. 2006). We also measured respiratory system impedance (*Z*_rs_) using a wave-tube system and modification of the forced oscillation technique (Sly et al. 2003).

We calculated airway resistance (*R*_aw_), tissue damping (G), and tissue elastance (H) by fitting the constant phase model to *Z*_rs_ data (Hantos et al. 1992) acquired at functional respiratory capacity (FRC) and also during a slow inflation-deflation manoeuvre from 0 to 20 cmH_2_O transrespiratory pressure (*P*_rs_). Specific lung compliance was calculated between *P*_rs_ = 8 cm/H_2_O and 3 cm/H_2_O on the deflationary arm, and %V10 was calculated at lung volume at *P*_rs_ = 10 cm/H_2_O divided by lung volume at *P*_rs_ = 20 cm/H_2_O (Limjunyawong et al. 2015).

The mice were then transferred to a small animal ventilator (flexiVent; SCIREQ, Montreal, Canada) for assessment of responsiveness to methacholine (MCh; acetyl β-methacholine chloride; Sigma-Aldrich, St Louis, MO) as previously described (Larcombe et al. 2011b).

#### Cellular inflammation and mediators in bronchoalveolar lavage

After lung function measurements were complete, bronchoalveolar lavage (BAL) fluid was collected from six males and six females from each treatment by connecting the tracheal cannula to a syringe and washing 0.5 mL of chilled saline in and out of the lungs three times. BAL samples were centrifuged for 4 min at 2000 rpm to separate supernatant and pellet. The total cell numbers were determined by staining the resuspended pellet with trypan blue and counting with a haemocytometer. Some cells were cytospun, stained with Rapid Diff (Australian Biostain Pty Ltd, Traralgon, Australia) and examined using light microscopy to obtain a differential cell count. Bio-Plex Pro^™^ Mouse Cytokine 23-plex Assays (Bio-Rad, Hercules, CA) were used to measure 23 mediators (eotaxin, granulocyte colony-stimulating factor (G-CSF), granulocyte macrophage-colony-stimulating factor (GM-CSF), interferon-gamma (IFN-γ), interleukins (IL-1α, IL-1β, IL-2, IL-3, IL-4, IL-5, IL-6, IL-9, IL-10, IL-12(p40), IL-12(p70), IL-13, IL-17 A), KC, monocyte chemoattractant protein-1 (MCP-1), macrophage inflammatory protein (MIP)−1α, MIP-1β, regulated on activation, normal T cell expressed and secreted (RANTES), and tumour necrosis factor alpha (TNF-α) in BAL supernatant in accordance with manufacturer’s instructions. Measurements below the limit of detection were replaced with a value half the lowest standard, for ease of statistical analysis.

#### Histology

Lung morphometry and airway composition were assessed in the male and female mice from each treatment that did not have BAL obtained. After lung function measurements were completed, the lungs were inflation-fixed by attaching the tracheal cannula to a column containing 4% formaldehyde at 10 cm H_2_O. Fixed lungs were embedded in paraffin and 5-µm thick sections were cut from the left lobe. Sections were stained with Masson’s Trichrome and Alcian Blue (with Nuclear Fast Red counterstain) for assessment of airway smooth muscle (ASM) mass, epithelial thickness, mean chord length (L_m_) and the proportion of mucus producing cells in the epithelium as previously described (Larcombe et al. 2017; Ramsey et al. 2013).

### RNA sequencing and analysis

The same mice from which we obtained bronchoalveolar lavage had pieces of lung from each lobe excised via blunt dissection. Samples were stored in RNAlater (Ambion, Life Technologies, Mulgrave, VIC, Australia) at 4 °C overnight, prior to RNAlater removal and then − 80 °C until use. Defrosted tissue was homogenised for 30 s in 350 µL RLT Plus buffer (QIAGEN, Hilden, Germany) using Precellys soft tissue homogeniser beads and Precellys 24 homogeniser (Bertin Technologies, Montigny-Le-Bretonneux, France), and total RNA was extracted using RNeasy Plus kit (QIAGEN, Hilden, Germany) as per the manufacturer’s instructions. The samples were sent to GenomicsWA for library preparation and sequencing to a depth of 20 million reads (Poly A Hs2 stranded RNASeq library preparation, iSeq QC and NovaSeq sequencing). The data were quality-assessed using FastQC (Andrews 2010) and aligned to *Mus musculus* mm10 genome reference using Hisat2 (Kim et al. 2019). Post-alignment quality control was performed with SAMstat (Lassmann et al. 2011). Sample QC was performed by examining pre- and post-alignment quality control data, density plots and principal component analysis plots. Two samples, one SA male and one SE male, were excluded from analysis due to low quality RNA and/or sequencing performance. Aligned data of remaining samples were analysed using DeSeq2 V1.28.1 (Love et al. 2014) to assess for differentially expressed genes (DEGs) between treatment groups, where male and female mice were assessed independently. Upstream regulator analysis was performed using Ingenuity Pathway Analysis (Qiagen), and results ranked by adjusted *p*-value. The 20 predicted upstream regulators with the lowest *p*-value were selected from each comparison for graphing. All analysis was performed on R statistical software V4.0.2 (R Development Core Team 2023) unless otherwise specified.

### Statistical analyses

Most data were analysed using SigmaPlot 14.0 (Systat Software Inc, San Jose, CA, USA). The groups were compared using two-way ANOVA, with sex and treatment as factors, or males and females were analysed separately via one-way ANOVA where appropriate, with Holm–Sidak post hoc tests. When required, data were transformed to satisfy the assumptions of normality and equal variance. Bioplex data were analysed using R Statistical Software (v4.2.0; R Core Team 2022) via multivariate general linear modelling methodologies with the families “Gamma(inverse/log)” and “gaussian(identity/log)” as best fits to the data. Data are reported as means ± SD. *p* < 0.05 was considered significant.

## Results

During the 12-week cigarette smoking period, a total of four mice (two males and two females) had to be euthanised as per our ethical obligations. These were 1 SS male for abnormal spinning behaviour (later found to have a brain lesion), 1 SA male for hunching and poor condition, 1 SS female which developed abdominal tumours, and 1 SA female for idiopathic weight loss. This left 11 mice in each of the above treatment groups and 12 in both SE treatment groups.

### Body weight

**Fig. 1:**
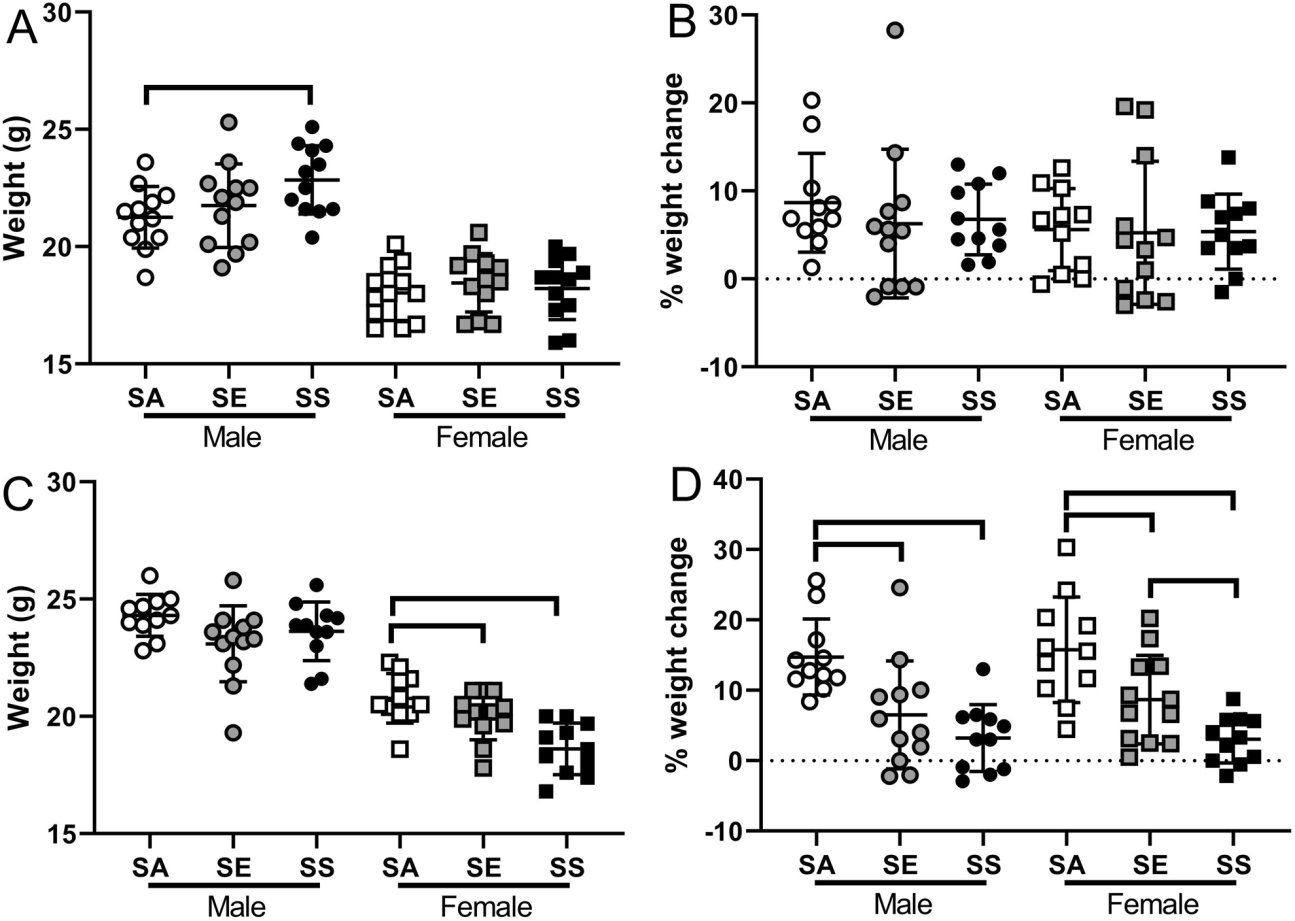
Effect of cigarette smoke and switching to e-cigarettes or quitting smoking on weight in male and female BALB/c mice. **A** weights prior to commencement of exposures, **B** percentage weight change after 12-weeks of cigarette smoke exposure, **C** weight on the day of study and **D** percentage weight change between the start and end of the experiment. Bars connect treatments that are significantly different within sex (*p* < 0.05). Data are individual mice with mean ± SD. *SA* smoke/air, *SE* smoke/e-cigarette, *SS* smoke/smoke

Body weight was measured prior to commencement of cigarette smoke exposures and then weekly throughout the duration of the study. Despite random allocation of mice to treatments SA males were significantly lighter than SS males at the beginning of the study (*p* = 0.046; Fig. 1A). There were no differences in starting weights between treatments for females (*p* = 0.709). On the day of study (i.e. 2 weeks after “switching”), there was no significant difference in weight in grams between treatments for male mice (*p* = 0.099), but for females, SS mice were significantly lighter than both SA and SE mice (*p* < 0.007 in both cases; Fig. 1C). Female SA and SE mice were not significantly different to each other (*p* = 0.095). Due to the slight difference in starting weight, weight gain as a percentage of starting weight was also used to assess differences between treatments. At the conclusion of the 12-week tobacco smoke exposure, there was no significant difference in percentage weight gain between treatments for male (*p* = 0.270) or female (*p* = 0.990) mice (Fig. 1B).

In terms of percentage increase from the commencement of the study, on the day of study SA male mice had increased in weight significantly more than both male SS and male SE mice (*p* < 0.009 in both cases; Fig. 1D), while male SS and SE mice were not significantly different (*p* = 0.204). Similarly, SA female mice had increased in weight significantly more than SS and SE mice (*p* < 0.016 in both cases), and SE females had increased in weight significantly more than SS mice (*p* = 0.031).

### Lung function and mechanics at functional residual capacity

There was a significant interaction between sex and treatment for lung volume at FRC (Supplementary Table 1), with male SE mice having significantly higher lung volumes than female SE mice (*p* < 0.001). There were no other significant differences (*p* > 0.065 in all cases). As there was a difference in lung volume, lung function at FRC is presented corrected for lung volume (i.e. specific *R*_aw,_ sR_aw_; specific G, sG and specific H, sH). At FRC, there was no effect of sex or treatment on sR_aw_ (*p* > 0.057 in both cases), and no interaction (*p* = 0.098). Nor was there any effect of treatment on sG (*p* = 0.806), however, there was a significant effect of sex on sG, with males having higher sG values than females (*p* = 0.041). There was no effect of sex or treatment on sH, and no interaction (*p* > 0.081 in all cases). There was a significant effect of sex (*p* < 0.001) and treatment (*p* = 0.006) on hysteresivity, with males having higher hysteresivity than females, and SS mice having higher hysteresivity than SA mice.

### Volume dependence of lung function

**Fig. 2:**
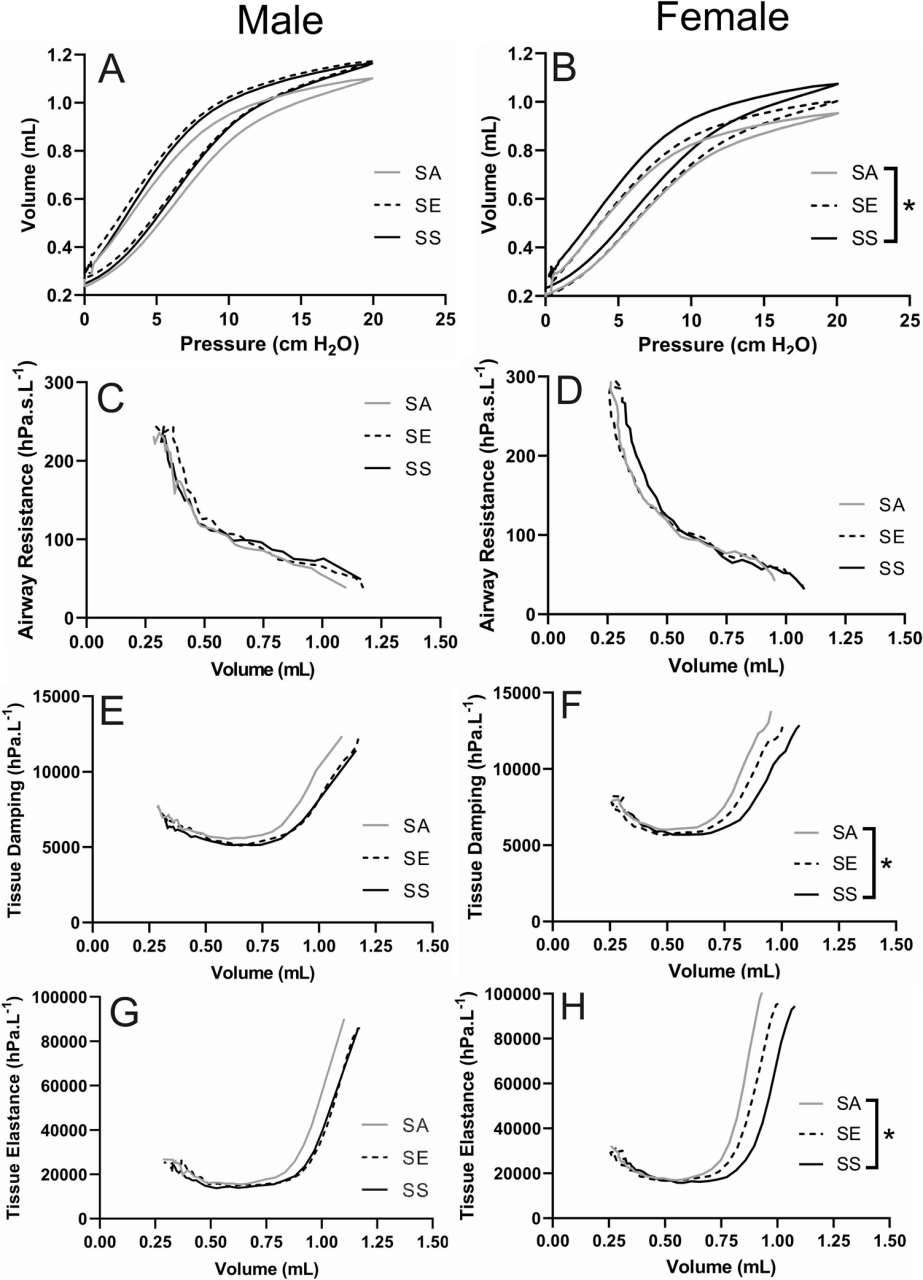
Pressure–volume loops for male (**A**) and female (**B**) mice and volume dependence of lung function for airway resistance (**C** male, **D** female), tissue damping (**E** male, **F** female) and tissue elastance (**G** male, **H** female). Data are group means. *N* = 11 for all groups, except female SA, male SA and male SE where *n* = 10. The differences between groups were analysed statistically at *P*_rs_ = 20 cm H_2_O for pressure volume loops, or a lung volume of 1.0 mL/0.8 mL (for male and female mice respectively) for other parameters. Curves are group averages. Bars connect treatments that are significantly different (*p* < 0.05). *SA* smoke/air, *SE* smoke/e-cigarette, *SS* smoke/smoke. Note different scales

There was a significant effect of treatment on lung volume at *P*_rs_ = 20 cm H_2_O for female mice (*p* = 0.028; Fig. 2B) but not male mice (*p* > 0.319 in all cases; Fig. 2A). Female SS mice had a significantly higher lung volume at *P*_rs_ = 20 cm H_2_O compared with female SA mice. There was no effect of treatment on specific compliance (*p* > 0.158 in all cases) or %V10 (*p* > 0.386) for either sex (Supplementary Table 2). We assessed differences in *R*_aw_, G and H at a lung volume of 1 mL for male mice, and 0.80 mL for female mice, those being approximately the highest lung volumes for which we had measurements for almost every individual (Fig. 2C–H). There was no effect of treatment on *R*_aw_, G or H at a lung volume of 1 mL for male mice (*p* > 0.112 in all cases), nor was there any effect of treatment on R_aw_ at a lung volume of 0.80 mL for female mice (*p* = 0.744). There was an effect of treatment on G and H at a lung volume of 0.80 mL for female mice (*p* < 0.023 in both cases). For both parameters, the value for air was significantly higher than that for smoke, but there was no difference between SE and either SA or SS.

### Responsiveness to methacholine

**Fig. 3:**
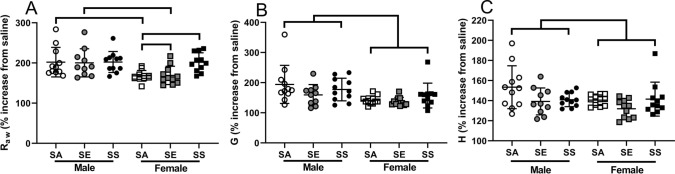
Airway resistance (**A**), tissue damping (**B**) and tissue elastance (**C**) shown as the percentage increase at 30 mg/mL from saline, of male and female BALB/c mice. Bars connect treatments that are significantly different (*p* < 0.05). Data are individual mice with mean ± SD. *SA* smoke/air, *SE* smoke/e-cigarette, *SS* smoke/smoke. Note different scales

Responsiveness to methacholine was analysed as the percentage increase from the saline aerosol (Fig. 3). There was a significant interaction between sex and treatment for airway resistance at the maximum dose of MCh (*p* = 0.049; Fig. 3A), whereby there was no effect of treatment on this parameter for males (*p* > 0.892 in all cases), but SS females were significantly more responsive than either SA (*p* = 0.003) or SE mice *(p* = 0.003), which were not different to each other (*p* = 0.955). Male SA and male SE mice were significantly more responsive than female SA and female SE mice respectively (*p* < 0.004 in both cases), however, there was no significant difference between male SS and female SS mice (*p* = 0.975).

In terms of tissue damping (G), there was no effect of treatment for either sex (*p* = 0.123), nor was there a significant interaction (*p* = 0.457; Fig. 3B). There was an overall effect of sex, with male mice having significantly higher G than female mice (*p* < 0.001). Similarly, there was an overall effect of sex on tissue elastance (H), with male mice having significantly higher H than female mice (*p* < 0.049; Fig. 3C). There was also a significant overall effect of treatment with SE mice having significantly lower H than SA mice (*p* = 0.012) regardless of sex. There was no difference between SS and SE (*p* = 0.208) or SS and SA (*p* = 0.172).

### Bronchoalveolar lavage cellular inflammation

**Fig. 4:**
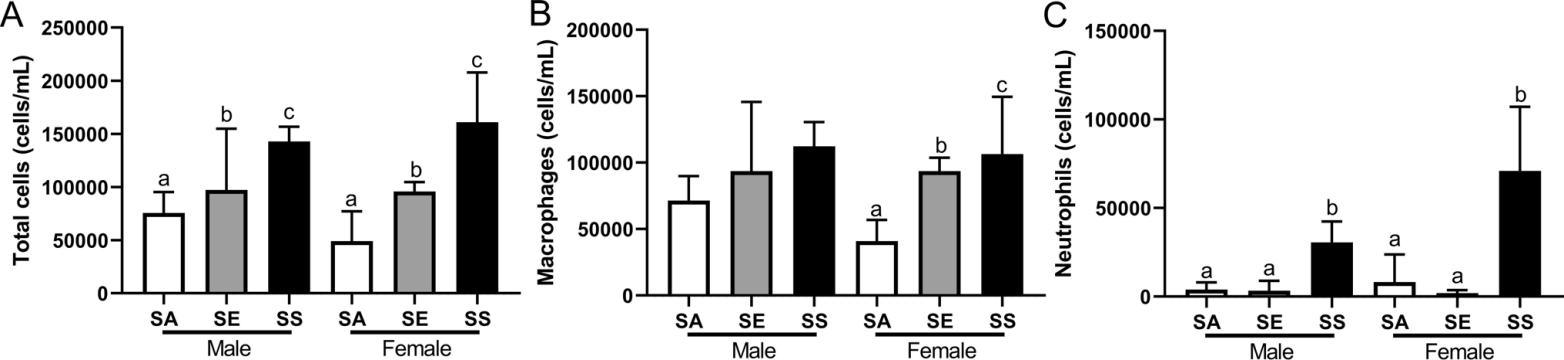
Total cells (**A**), macrophages (**B**) and neutrophils (**C**) in the bronchoalveolar lavage of male and female BALB/c mice. Different letters indicate significant differences between treatments within sex (*p* < 0.05). Data are mean ± SD. *SA* smoke/air, *SE* smoke/e-cigarette, *SS* smoke/smoke. Note different scales

There was no significant effect of sex on total cellular inflammation in bronchoalveolar lavage (*p* = 0.559) and no interaction between sex and treatment (*p* = 0.160), however, there was a significant overall effect of treatment, with SS mice having significantly more cells than either SE or SA mice (*p* < 0.001 in both cases; Fig. 4A). SA mice also had significantly fewer total cells in their lavage than SE mice (*p* = 0.007). Cellular inflammation was dominated by macrophages and neutrophils, with trace numbers of lymphocytes and eosinophils detected in a small proportion of mice. There was significant interaction between sex and treatment for macrophage numbers (*p* = 0.050; Fig. 4B). SA female mice had significantly fewer macrophages than female SE or SS mice (*p* < 0.001 in both cases), while female SS and SE mice were not significantly different (*p* = 0.817). Although the same overall trend was present, there was no statistically significant effect of treatment for male mice (*p* > 0.076 in all cases). There was a significant effect of treatment on neutrophil numbers whereby SS mice had significantly higher numbers of neutrophils compared to both other treatments (*p* < 0.001; Fig. 4C). There was no effect of sex (*p* = 0.807) and no interaction (*p* = 0.268).

### Mediators in BAL supernatant

**Fig. 5:**
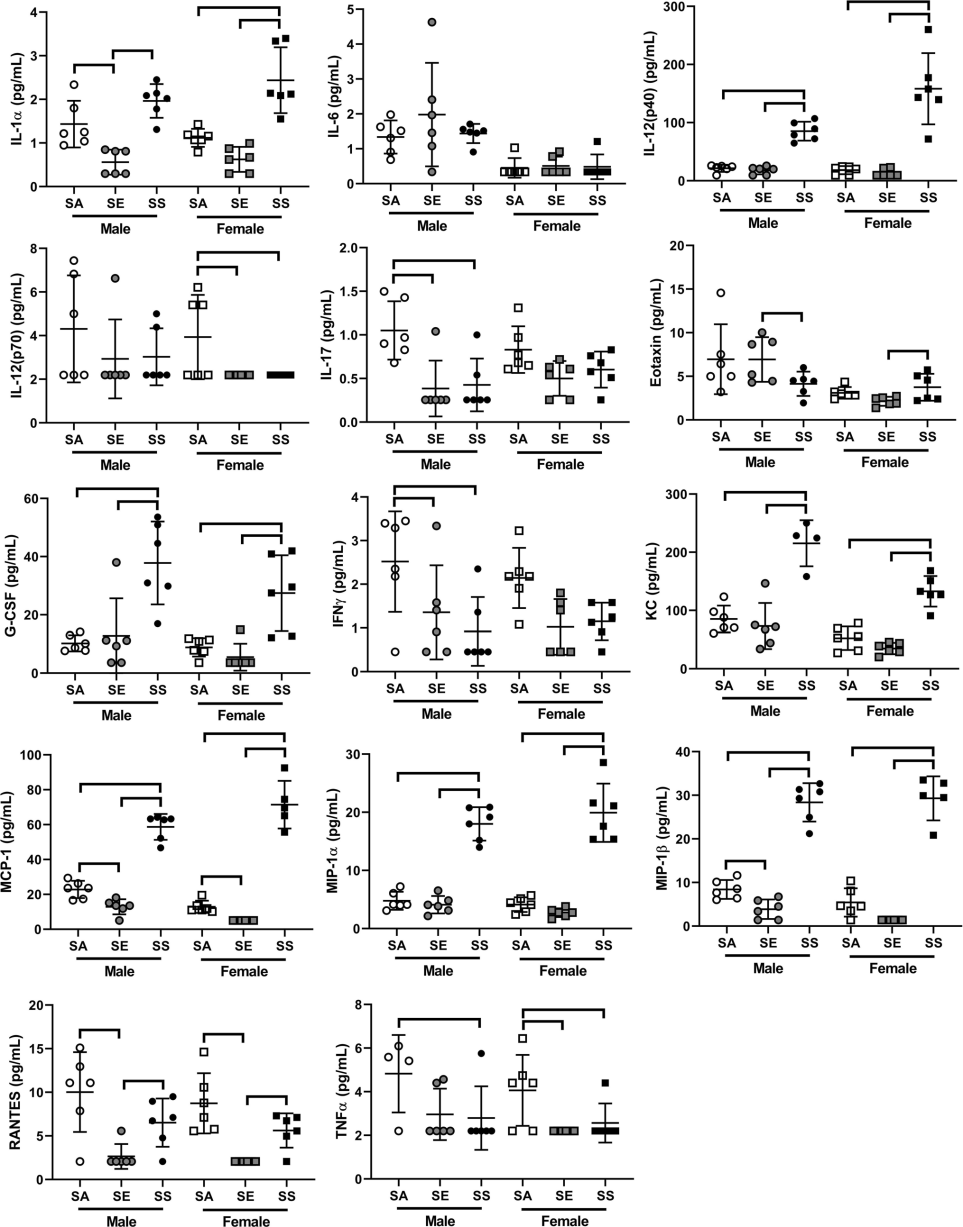
Mediators in bronchoalveolar lavage of male and female BALB/c mice. Bars connect treatments that are significantly different (*p* < 0.05) within sex. Data are individual mice with mean ± SD. *SA* smoke/air, *SE* smoke/e-cigarette, *SS* smoke/smoke. Note different scales

Of the 23 mediators assessed, 14 were detected in BAL supernatant at levels above the limit of detection of the kit—IL-1α, IL-6, IL-12(p40), IL-12(p70), IL-17, eotaxin, G-CSF, IFN-γ, KC, MCP-1, MIP-1α, MIP-1β, RANTES and TNFα (Fig. 5). For simplicity, males and females were analysed separately. There was no effect of treatment on IL-6 for either male (*p* > 0.802 in all cases) or female (*p* > 0.709 in all cases) mice, however, there were complex effects of treatment for all other mediators, which in many cases differed by sex. In many cases, significant differences were identified between SS and SA and/or SE mice. For IL-12(p40), G-CSF, KC and MIP-1α, SS mice had significantly higher levels than both SA and SE mice for both males and females (*p* < 0.05 in all cases). This was also true for MIP-1β, for which male SE mice also had significantly lower MIP-1β compared with male SA mice. A similar pattern was seen for MCP-1 (highest levels in SS mice, lowest in SE mice). This “suppression” was seen for a number of mediators, whereby SE mice of one, or both, sexes, had significantly lower levels than SA mice; IL-1α, IL-17, RANTES, and TNFα.

### Lung structure

There were few statistically significant effects of treatment on parameters of lung structure (Supplementary Table 3). There was no effect of sex or treatment on lumen area (*p* > 0.299 in both cases), airway smooth muscle mass (*p* > 0.568 in both cases) or the perimeter of the basement membrane (*p* > 0.187 in both cases). There was also no statistically significant effect of treatment on chord length (*p* = 0.068), although there was a trend of decreasing chord length from SS to SE to SA mice in both sexes. Mean chord length was 11.0% longer in male-SS compared with male-SA and 21.6% longer in female-SS compared with female-SA, with SE mice in between for both sexes. There was a significant effect of treatment on mucus producing cells, with SS mice having significantly more per unit length of basement membrane, compared with SE mice (*p* = 0.019). There was no effect of sex, or any interaction between sex and treatment for this parameter (*p* > 0.114 in both cases).

### Differential gene expression analysis

**Fig. 6:**
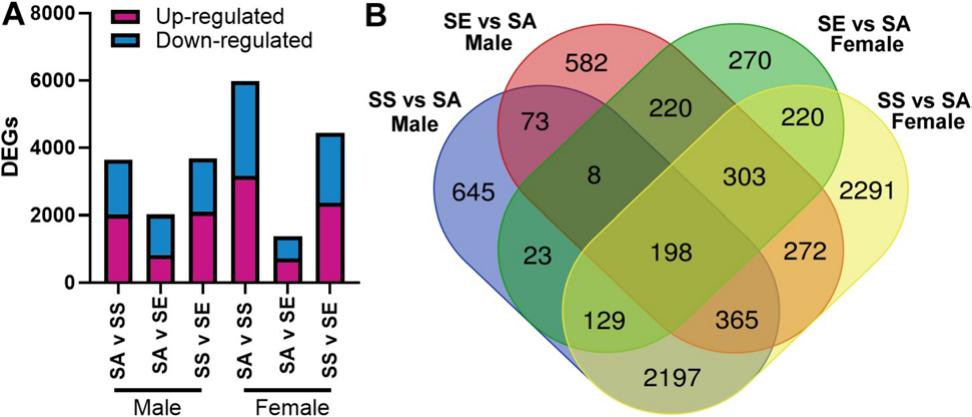
Differentially expressed genes (DEGs). **A** Total number of DEGs compared to Air controls. Red colouration indicates up-regulated, or increased, expression and blue down-regulated, or decreased, expression. **B** Venn-diagram of unique and shared number of DEG’s for SS and SE male and female mice compared to their respective SA controls. Drawn using http://bioinformatics.psb.ugent.be/webtools/Venn/

Aligned data from *n* = 5 for SA male, SA female and SE male and *n* = 6 SE female, SS male and SS female were analysed for differential gene expression (Fig. 6). Female SS mice showed the largest response compared to both SA and SE exposure (5975 and 4437 DEGs, respectively) whereas these exposures in male mice resulted in 3638 and 3680 DEGs, respectively (Fig. 6A). The female SS group also had the largest number of unique DEGs (2291) when compared to their respective SA group, whereas every other comparison to air control groups had less than 1000 unique DEGs (Fig. 6B). In contrast, the male mice showed the largest response to e-cigarette aerosol exposure with 2021 DEGs between SE male and SA males compared to the females 1371 DEGs, as well as 582 unique DEGs compared to the females 270.

Upstream regulator analysis was performed to infer master regulators operating upstream of the analysed transcriptional changes. The top 20 drivers ranked by *p* value are shown for each comparison within male and female mice (Supplementary Fig. 1). For both male and female SS mice, the majority of top ranked drivers were predicted to be activated and predicted regulators were mostly related to the immune response, both innate and adaptive, including *CSF2*, *IFNG*, *IL4*, *IL6*, *TNF*, *MYD88*, *IL33*, *IL1B*, and *IL1 A*. This was in contrast to the male and female SE groups where the majority of top ranked upstream regulators were predicted to be inhibited. These genes mostly related to DNA damage responses, cell cycle regulation and growth factors including *FOXM1*, *PTGER2*, *CKAP2L*, *MXD1*, *VEGF*, *TCF3*, *CCND1*, *LIN9*, and *NUPR1* (Supplementary Fig. 1 C, D). Of note, *PTGER2*, a top ranked driving gene predicted to be activated in the SS female mice, was predicted to be inhibited in both SE male and SE female mice. A similar pattern was found with *CEBPB*, which was predicted to be activated in both SS male and female mice but inhibited in SE female mice. Whereas the opposite pattern was found in *CDKN1A* which was predicted to be inhibited in both SS male and SS female mice but activated in SE female mice. All three of these genes are in the top 10 ranked DEGs when comparing SS male and SE male gene expression and the top five ranked DEGs when comparing SS female and SE female mice (Supplementary Fig. 1E, F).

## Discussion

The results of this study indicate that switching to e-cigarette use after long-term tobacco cigarette exposure (as opposed to continued smoking), leads to improvements in aspects of respiratory health in mice. In general, the improvements measured were less pronounced in mice that switched to e-cigarettes, compared with those that quit cigarette smoking altogether.

A key outcome of this study was that there were clear sex differences in outcomes, with most impacts being statistically significant in female mice only. This outcome is consistent with the literature where it has previously been shown that female mice develop cigarette-smoke induced COPD-like pathologies at a faster rate than male mice (March et al. 2006; Tam et al. 2016) and that effects are dose-dependent (March et al. 2006). This increased susceptibility has been associated with increased oxidative stress and TGF-β1 signalling (Tam et al. 2016). Female sex hormones also play a role, as ovariectomized female mice develop pathologies at a similar rate to male mice. Similarly, epidemiological studies indicate a greater susceptibility to cigarette-smoke induced COPD in women compared with men (Langhammer et al. 2003; Viegi et al. 2001). In our study, the 14 weeks of cigarette smoke exposure for SS mice may have been sufficient to elicit a severe enough pathology in female mice for differences between switching to e-cigarettes to be more noticeable compared with male mice.

An initial finding of this study was that quitting cigarette smoke exposure resulted in significant weight gain in both sexes (Fig. 1). This finding was not unexpected due to the known appetite suppressant effect of nicotine (Mineur et al. 2011), and the knowledge that smoking cessation is linked with higher relative weight in humans (Rasky et al. 1996). We have also previously shown that exposure to cigarette smoke, or e-cigarette aerosol containing nicotine results in slower weight gain in adolescent mice (Larcombe et al. 2017). Other researchers have shown that exposure to e-cigarette aerosols containing nicotine impairs weight gain in mice (Moore et al. 2023), thus it was somewhat surprising that, for female mice, switching to e-cigarettes (SE) also led to an increase in body weight compared with continued smoking (SS). This could potentially be due to our “whole-body” exposure protocol, whereby e-cigarette aerosol would deposit on the fur of the mice and be ingested as they groom. E-liquid is primarily glycerol and propylene glycol by volume, both of which have a sweet taste and a caloric component of around 4 cal per gram. It could also be a dosing effect (i.e. e-cigarette exposed mice received less nicotine than cigarette smoke exposed mice), although we attempted to match our “puffing” regimes between treatments. Importantly, others have shown weight gain impairment in e-cigarette exposed mice is not necessarily due to the appetite-suppressant effects of nicotine as it occurs in mice exposed to nicotine-free e-liquid (McGrath-Morrow et al. 2015).

We identified significant effects of treatment on various parameters of lung function (Supplementary Tables 1 and 2 and Figs. 2 and 3). While there was no biologically relevant effect of treatment at functional residual capacity, the volume dependence of lung function, and responsiveness to MCh were both impacted by treatment. Importantly, the effects seen were almost exclusively seen in female mice. A key finding was that quitting cigarette smoking entirely led to a decrease (or lesser increase) in lung volume at *P*_rs_ = 20 cm H_2_O (Fig. 2A and B). This was seen for both sexes but was only statistically significant for female mice. Male mice that switched from cigarette smoking to e-cigarettes were statistically indistinguishable from those that continued smoking for this parameter. It is well known that long-term cigarette smoke exposure (Dobric et al. 2022; Dubois-Deruy et al. 2020; Serré et al. 2021) leads to reduced elastic recoil and alveolar collapse/destruction in mice, which manifests in an upward shift in the PV-loop (Dobric et al. 2022). Therefore, this “decrease” could also be interpreted as mice which continued to smoke (SS) having continued lung damage and hence an even higher lung volume at *P*_rs_ = 20 cm H_2_O compared with switching or quitting. Female mice that switched to e-cigarettes (SE) were approximately halfway between the other two treatments (although not significantly different to either), while male SE mice were indistinguishable from male SS mice for this parameter. This indicates that switching to e-cigarettes leads to pathology consistent with this physiological change. It is also consistent with findings of recent studies, which show that exposing mice to e-cigarette aerosols for 8–9 weeks can lead to structural and function changes concordant with emphysema (Rodriguez-Herrera et al. 2023; Zhao et al. 2024)—such that 2 weeks of e-cigarette aerosol exposure had negative effects on the volume dependence of lung function, but these effects were less severe than those elicited by continued smoking.

Quitting cigarette smoking also changed the volume dependence of tissue damping and tissue elastance for female mice. Perhaps counterintuitively, the trend was for higher G and H at a lung volume of 0.8 mL for female SS mice compared with female SA mice (Fig. 2)—although there were no differences in these parameters at FRC. It might be expected that both G and H would be lowest in SS mice, due to the known pathophysiology of more “empty space” in the alveolar structure in emphysematous mice (Devos et al. 2017), however, Copot et al. (2017) showed that in mild (COPD-GOLD II) disease, tissue damping is substantially higher than control. The authors surmise that parenchymal/alveolar destruction inherently means a loss of structural components responsible for lung elastance (e.g. collagen and elastin). This therefore leads to increased elastance. Further, they suggest that damping “will increase due to increased tissue density in places where alveolar walls are not broken”. In our model, the cigarette-smoke induced phenotype was mild, so may be similar to COPD-GOLD II in these respects. Any effects on lung function at FRC may have been too mild to measure using our techniques, with treatment differences only becoming apparent at higher lung volumes.

The mild effects we saw in the volume dependence of lung function were reflected in lung structural parameters. While we did not see a statistically significant effect of treatment on chord length, there was a clear trend of SS mice having longer *L*_m_, followed by SE, followed by SA (Supplementary Table 3). This finding supports the well-established knowledge that long-term cigarette smoke exposure increases chord-length in mice (Shu et al. 2017). It is somewhat counter to the finding of two recent publications, however, whereby 8–9 weeks of e-cigarette aerosol exposure in mice resulted in even greater chord lengths than mice exposed to traditional cigarettes for the same duration (Rodriguez-Herrera et al. 2023; Zhao et al. 2024). In our study, the switch to e-cigarettes appears to have slightly attenuated the ongoing alveolar destruction caused by continued smoking. This is also reflected in the lack of a statistically significant effect of treatment on compliance. Like *L*_m_, there was a trend of increasing compliance from SA < SE < SS, with SS mouse compliance being 12.6% higher than SA mice in females, and 6.8% higher in males. This statistically non-significant result may be due to insufficient statistical power, as similar smoke exposure protocols often show that long-term cigarette smoke exposure in mice results in increased compliance (Fricker et al. 2014; March et al. 2006). That said, Foronjy et al (2005) found that while up to 1 year of cigarette smoke exposure in mice led to emphysematous structural changes, there was no associated increase in lung compliance (Foronjy et al. 2005).

Responsiveness to MCh was another parameter where effects were seen for female mice, but not males. The female SS mice were more responsive than either SE or SA mice, particularly with respect to airway resistance, suggesting that replacing cigarettes with e-cigarettes, or quitting entirely has some beneficial effects on airway hyper-reactivity. This has important implications from a number of perspectives. First, as smoking prevalence in asthma is similar to that of the general population, even in cases of severe asthma (Katsaounou et al. 2019), and as there is a view that e-cigarette use is less harmful than cigarette smoking, smoking asthmatics may consider switching to e-cigarettes. There is even support for this switch by some researchers and clinicians (Polosa et al. 2014, 2016a), although these studies are old, and the authors exhibit substantial conflicts of interest. Our data show, that in female mice, such a change had no statistically significant beneficial effect on airway hyper-responsiveness (SS and SE female mice were not significantly different to each other for *R*_aw_ at 30 mg/mL MCh; Fig. 3A). Further, e-cigarette use has been associated with initiation and exacerbation of asthma (Cho and Paik 2016; Deshpande et al. 2020; Schweitzer et al. 2017), and may even lead to second-hand exacerbations in non-users (Bayly et al. 2019). A review on e-cigarettes concluded that “the negative effects of e-cigarettes may be exaggerated in people with asthma” (Clapp and Jaspers 2017), while another concluded that “asthmatic patients should avoid using EC (electronic cigarettes) (Kotoulas et al. 2021).

With respect to cellular inflammation in the bronchoalveolar lavage, we saw a range of exposure related effects, which again were more overt in female mice (Fig. 4). Overall, SS mice had the highest number of total cells in their BAL, followed by SE, with SA mice having the fewest. This pattern was not unexpected, as long-term cigarette smoke exposure is known to elicit an inflammatory response in mice, primarily consisting of macrophages and neutrophils (D'hulst et al. 2005; Larcombe et al. 2017). It was also not surprising that quitting cigarette smoke exposure (SA) resulted in total cell numbers returning to levels similar to seen in naïve BALB/c mice in our laboratory (Larcombe et al. 2017), and an almost complete abolition of neutrophilia. Of interest, however, was the statistically significant reduction in total cells in the BAL in SE mice, but this reduction not being a “return to normal”. In our previous study (Larcombe et al. 2017), we found no increase in cells in the BAL of mice exposed to e-cigarette aerosols for 8 weeks. However, other studies have shown that e-cigarette aerosol exposure elicits an inflammatory response in mouse lungs [reviewed in (Masso-Silva et al. 2021a)]. Therefore, for this parameter, a switch to vaping was beneficial compared with continued smoking, but not as beneficial as quitting entirely.

We also found a range of complex effects of sex and treatment on levels of mediators in bronchoalveolar lavage (Fig. 5). There were, however, several overarching patterns in response. One was for certain mediators to be highest in SS mice regardless of sex (e.g. IL-12(p40), G-CSF, KC, MCP-1, MIP-1α, MIP-1β). These are well known macrophage and neutrophil chemotactic mediators (Cooper and Khader 2007; Deshmane et al. 2009; Kim et al. 2020; Maurer and Von Stebut 2004), so it is not surprising that they are highest in the BAL of SS mice. We also saw a reduction in some mediators in SE mice compared with SA mice (e.g. IL-1α, IL-17, MIP-1α, MCP-1, RANTES). Amongst other functions, these mediators are important in defence against viral, bacterial and/or fungal infections (Appay and Rowland-Jones 2001; Dinarello 2009; Mills 2023) suggesting that SE mice may have impaired responses to these insults. Chronic e-cigarette aerosol exposure has previously been shown to impair the immune function of mouse lungs and increase susceptibility to infectious insults (Masso-Silva et al. 2021b; Sussan et al. 2015). It is clear from these results that chronic cigarette smoking has a wide range of adverse impacts on mouse lungs and that switching to e-cigarettes elicits responses that are different to quitting entirely. The apparent suppression of some key mediators in SE mice is a topic that warrants further exploration.

The greatest number of DEGs, as well as the greatest number of unique DEGs, was observed between SS and SA female mice. In contrast, the smallest number of DEGs was observed between SE and SA female mice, with SS and SE female mice comparisons showing a DEG count in the middle of these two analyses. This again suggests that female mice that switch to e-cigarettes display improvements but not as much as mice that quit smoking altogether. Similarly, this pattern is not as overt in the male mice, where SS and SE and SS and SA comparisons show similar number of DEG’s, and only the SE and SA comparison shows a decrease in the number of DEGs. With upstream regulator analysis, the majority of predicted activated genes were related to the immune response for both male and female SS mice. Of note, while *IFNG*, *IL6* and *TNF* were predicted to be activated in the upstream regulator analysis, actual measurements in the BAL showed either a significant decrease or no change. This could be explained by the cytokines in the BAL being “used up” and the RNA sequencing data and upstream regulator analysis capturing the corrective response (Young et al. 2020).

In comparison, upstream regulator analysis in the SE male and female mice predicted a majority of inhibited drivers, related mostly to DNA damage responses, cell cycle regulation and growth factors. This is difficult to interpret as some of the predicted inhibited genes are negative regulators of downstream effects (e.g. *CCND1* (2024)) but could indicate a repair response in the SE mice, either to counteract the damage of the long term smoke exposure or from e-cigarette exposures causing its own damage, particularly to DNA (Yu et al. 2016). There were also three genes that were predicted to have opposite responses in the SS and SE mice. *PTGER2*, predicted to be activated in SS female mice and inhibited in SE male and female mice, is a gene that encodes a receptor for prostaglandin E. *CEBPB,* predicted to be activated in SS male and female mice and inhibited in SE female mice, encodes CCAAT/enhancer binding protein beta. Both of these genes regulate a wide range of biological processes, but share an effect of regulating inflammation (Ren et al. 2023; Sugimoto and Narumiya 2007). As the BAL immune cell and mediator measurements indicate a general trend of increased inflammation in the SS mice and potentially supressed inflammation in the SE mice, these genes could be regulators of the observed differences. In contrast, *CDKN1A* is predicted to be activated in SE female mice and inhibited in SS male and female mice. It codes for cyclin-dependent kinase inhibitor 1, which plays an important role in regulating cell cycle suppression in response to DNA damage (Karimian et al. 2016). This may further indicate DNA damage caused by e-cigarette exposure, although more research is needed to confirm.

This study must be interpreted in terms of its strengths and limitations. Its key strength is that, to the best of our knowledge, it presents the first in-depth research investigating the effects of switching to e-cigarette use after chronic smoke exposure. We provide a detailed physiological, immunological and molecular assessment of the effects of this switch. We acknowledge the limitations of our model, including the dose/duration of exposure, and the choice of e-liquid. As previously mentioned, 12 weeks of cigarette smoke exposure may not have been sufficient to elicit severe respiratory disease in this study, even though it resulted in a distinct phenotype. Similarly, the 2-week “switch” or “quit” time may have been too short for all effects of e-cigarette exposure, or quitting, to be fully realised. Further, we did not include a “dual-use” exposure group, despite the prevalence of dual-use (Carpenter et al. 2023) and the likelihood of dual-use being as harmful, or more harmful than exclusive cigarette smoking (Pisinger and Rasmussen 2022). We also did not investigate the potential for different flavours to impact outcomes. These limitations provide avenues for future research. Regardless, in conclusion, we have shown that switching to e-cigarettes after long-term cigarette smoke exposure has beneficial effects on respiratory health in mice (compared with continued smoke exposure), but that these improvements are not as overt as those seen when mice quit smoking and are only given air. Importantly, switching to e-cigarettes was not harm-free, and induced a range of molecular and immunological effects that are indicative of immune suppression. Our study shows that quitting cigarette smoking is the best option. More research is required to fully assess the long-term impacts of e-cigarette use after chronic cigarette smoke exposure.

## Supplementary Information

Below is the link to the electronic supplementary material.

Supplementary file1 (DOCX 273 KB)

Supplementary file2 (JPG 1155 KB)

## Data Availability

The data underlying this article will be shared on reasonable request to the corresponding author.
