# Supplementary material for: Partial amelioration of a chronic cigarette-smoke-induced phenotype in mice by switching to electronic cigarettes
Source: Arch Toxicol. 2025 Apr 18;99(7):3007–21. doi: 10.1007/s00204-025-04055-7 (PMC12198299; doi:10.1007/s00204-025-04055-7)
Supplement: Supplementary file 1 — Supplementary file1 (DOCX 273 KB) [file 204_2025_4055_MOESM1_ESM.docx]

Supplementary Results

**Supplementary Table 1** – Lung volume and lung function at functional residual capacity. *indicates significant effect of sex for a specific treatment, # indicates an overall difference between males and females.

| Treatment (n) | Lung volume (mL) ± SD | sR_aw_ at FRC (hPa.s^-1^) ± SD) | sG at FRC  (hPa) ± SD | sH at FRC  (hPa) ± SD | Hysteresivity |
| --- | --- | --- | --- | --- | --- |
| Male-SA (11) | 0.24 ± 0.04 | 79.5 ± 16.6 | 1815 ± 288^#^ | 6852 ± 883 | 0.265 ± 0.019 |
| Male-SE (12) | 0.28 ± 0.05* | 92.3 ± 12.3 | 1954 ± 236^#^ | 7170 ± 706 | 0.273 ± 0.018 |
| Male-SS (11) | 0.25 ± 0.02 | 87.7 ± 17.8 | 1796 ± 178^#^ | 6438 ± 849 | 0.280 ± 0.018 |
| Female-SA (11) | 0.21 ± 0.03 | 79.1 ± 12.5 | 1708 ± 275^#^ | 6949 ± 1030 | 0.246 ± 0.016 |
| Female-SE (11) | 0.20 ± 0.03* | 78.7 ± 14.8 | 1628 ± 262^#^ | 6419 ± 967 | 0.254 ± 0.011 |
| Female-SS (11) | 0.23 ± 0.04 | 94.9 ± 21.4 | 1830 ± 307^#^ | 7008 ± 1354 | 0.263 ± 0.015 |

**Supplementary Table 2** – Lung compliance, specific lung compliance, %V10 and lung volume at P_rs_=20cm H_2_O.

| Treatment (n) | Compliance | Specific Compliance | %V10 | V at  P_rs_=20cmH2O |
| --- | --- | --- | --- | --- |
| Male-SA (11) | 0.073 ± 0.010 | 0.145 ± 0.019 | 0.861 ± 0.014 | 1.101±0.139 |
| Male-SE (12) | 0.075 ± 0.012 | 0.131 ± 0.014 | 0.870 ± 0.016 | 1.173 ± 0.182 |
| Male-SS (11) | 0.078 ± 0.007 | 0.144 ± 0.017 | 0.866 ± 0.011 | 1.163 ± 0.069 |
| Female-SA (11) | 0.063 ± 0.005 | 0.146 ± 0.023 | 0.863 ± 0.013 | 0.953± 0.084 |
| Female-SE (11) | 0.065 ± 0.004 | 0.148 ± 0.014 | 0.857 ± 0.018 | 1.004 ± 0.066 |
| Female-SS (11) | 0.071 ± 0.008 | 0.143 ± 0.014 | 0.866 ± 0.014 | 1.074 ± 0.103 |

**Supplementary Table 3** – Lung structure parameters of male and female BALB/c mice. Data are present as mean ± SD. SA = smoke/air, SE = smoke/e-cigarette, SS = smoke/smoke.

| Treatment (n) | L_m_ (µm) | P_bm_ (µm) | ASM  (√area/P_bm_) | Lumen, (√area/Pbm) | Mucus producing cells |
| --- | --- | --- | --- | --- | --- |
| Male-SA (5) | 25.48 ± 1.39 | 3232 ± 752 | 0.040 ± 0.008 | 0.186 ± 0.043 | 0.014 ± 0.011 |
| Male-SE (5) | 27.55 ± 2.11 | 2863 ± 521 | 0.043 ± 0.008 | 0.206 ± 0.030 | 0.011 ± 0.002 |
| Male-SS (5) | 28.29 ± 2.92 | 3034 ± 379 | 0.040 ± 0.005 | 0.225 ± 0.032 | 0.018 ± 0.006 |
| Female-SA (5) | 25.00 ± 2.03 | 3094 ± 129 | 0.041 ± 0.004 | 0.213 ± 0.018 | 0.011 ± 0.008 |
| Female-SE (6) | 29.63 ± 11.75 | 3401 ± 1166 | 0.042 ± 0.010 | 0.187 ± 0.037 | 0.005 ± 0.003 |
| Female-SS (4) | 30.41 ± 4.94 | 3693 ± 632 | 0.037 ± 0.008 | 0.178 ± 0.028 | 0.015 ± 0.004 |

Values are means ± SD. ASM = airway smooth muscle; L_m_ = mean linear intercept; P_bm_ = perimeter of basement membrane. Mucus producing cells are the number of positive cells divided by P_bm_.


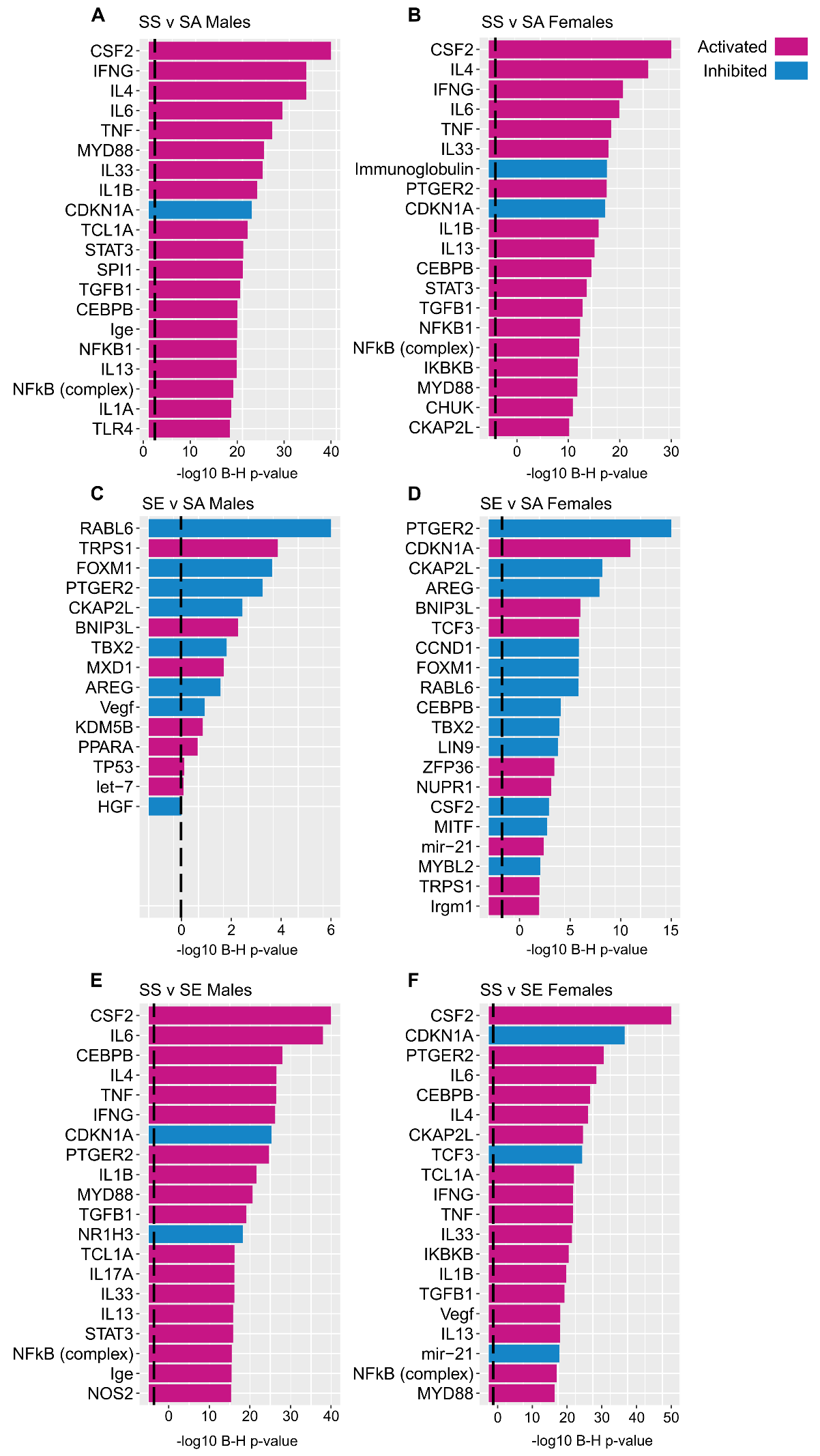


**Supplementary Figure 1**: The 20 top ranked predicted transcriptional drivers by p-value for each comparison after upstream regulator analysis. Red colouration indicates predicted up-regulation, blue colouration indicates predicted down-regulation. A) SS vs SA males, B) SS vs SA females, C) SE vs SA males, D) SE vs SA females, E) SS vs SE males, F) SS vs SE females. n=6 per group except for SA male, SA female and SE male where n=5.
