# Supplementary figures and images for: Partial amelioration of a chronic cigarette-smoke-induced phenotype in mice by switching to electronic cigarettes
Source: Arch Toxicol. 2025 Apr 18;99(7):3007–21. doi: 10.1007/s00204-025-04055-7 (PMC12198299; doi:10.1007/s00204-025-04055-7)

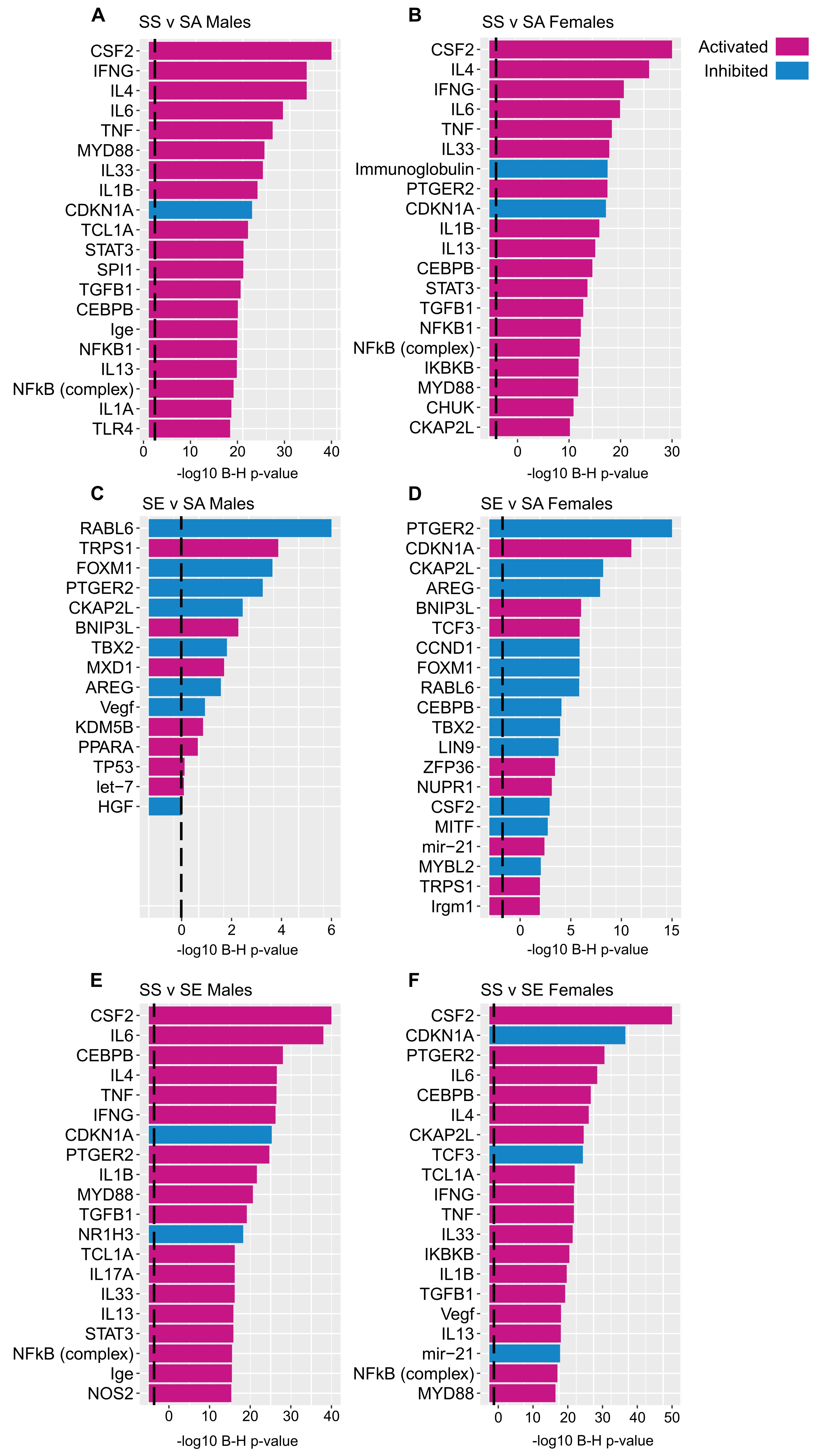

Supplement: Supplementary file 2 — Supplementary file2 (JPG 1155 KB) [file 204_2025_4055_MOESM2_ESM.jpg]
